# Longitudinal Trajectories and Inter-parental Dynamics of Prairie Vole Biparental Care

**DOI:** 10.3389/fevo.2018.00073

**Published:** 2018-06-05

**Authors:** Forrest D. Rogers, Mijke Rhemtulla, Emilio Ferrer, Karen L. Bales

**Affiliations:** 1 Department of Psychology, University of California, Davis, Davis, CA, United States; 2 California National Primate Research Center, University of California, Davis, Davis, CA, United States

**Keywords:** monogamy, prairie vole, biparental care, latent growth model, structural equation model

## Abstract

For altricial mammalian species, early life social bonds are constructed principally between offspring and their mothers, and the mother-offspring relationship sets the trajectory for offspring bio-behavioral development. In the rare subset of monogamous and biparental species, offspring experience an expanded social network which includes a father. Accordingly, in biparental species fathers also have the potential to influence trajectories of offspring development. Previous semi-natural and laboratory study of one monogamous and biparental species, the prairie vole (*Microtus ochrogaster*), has given insight into the role that mothers and fathers play in shaping behavioral phenotypes of offspring. Of particular interest is the influence of biparental care in the development of monogamous behavior in offspring. Here, we first briefly review that influence. We then present novel research which describes how parental investment in prairie voles changes across sequential litters of pups, and the extent to which it is coordinated between mothers and fathers. We use approximately 6 years of archival data on prairie vole parenting to investigate trajectories and inter-parent dynamics in prairie vole parenting. We use a series of latent growth models to assess the stability of parental investment across the first 4 l. Our findings suggest that prairie voles display sexually dimorphic patterns of change in parental behavior: mothers’ investment declines linearly whereas fathers’ pattern of change is characterized by initial decline between litters 1 and 2 with subsequent increase from litters 2 to 4. Our findings also support a conclusion that prairie vole paternal care may be better characterized as compensatory—that is, fathers may compensate for decline in maternal investment. Opposing trends in investment between mothers and fathers ultimately imply stability in offspring investment across sequential litters. These findings, combined with previous studies, generate a hypothesis that paternal compensation could play an important role in maintaining the development of monogamous behavioral phenotypes in individual offspring and across cohorts of those offspring. Understanding longitudinal and inter-individual dynamics of complex social behaviors is critical for the informed investigation of both proximate and ultimate mechanisms that may subserve these behaviors.

## INTRODUCTION

Social bonds formed in early life are particularly influential in the development of adult social behaviors; early life experiences have incredible potential to shape individual variation (Meaney, 2001). Of particular importance to bio-behavioral development is the role of the mother and the social relationship between her and her offspring in altricial mammals. Of the 3–5% of monogamous mammalian species (Kleiman, 1977), many display biparental care (infant care by both parents), and the dynamics of this parenting are of particular interest given the high frequency of biparental care in humans. It is generally agreed that paternal care (and therefore biparental care) emerged after monogamy (Lukas and Clutton-Brock, 2013, 2014; Opie et al., 2013, 2014). Biparental care is demonstrated in a range of mammals, including but not limited to rodents (e.g., prairie voles, California mice, white-footed mice, etc.) (McGuire and Bemis, 2007), canids (e.g., Maned wolves, red foxes, African wild dogs, etc.) (Malcolm, 1985), and primates (e.g., titi monkeys, common marmoset, Goeldi’s monkey, humans, etc.) (Schradin et al., 2003; Fernandez-Duque et al., 2009; Bales and Jarcho, 2013).

Under conditions of biparental care, developing young have an expanded early life social network, allowing mothers to distribute the labor of parental investment and allowing offspring the benefit of a slower and safer development (Hrdy, 2009). In biparental rodent species, offspring may benefit from improved rates of survival and growth (McGuire and Bemis, 2007). Among biparental canids, young benefit from increased defense and provisioning (Malcolm, 1985). In biparental primates, particularly arboreal New World monkeys, fathers play an important role in the transport of offspring (Tardif, 1994; Fernandez-Duque et al., 2009). The relative role of fathers can be quite extensive, such as in the case of the titi monkey (*Callicebus moloch*), in which fathers do the majority of infant transport and in which infants show extensive distress upon separation from the father, but not mother (Hoffman et al., 1995).

The way in which maternal and paternal investment changes across time (i.e., across sequential births or litters) varies by sex and species (McGuire and Bemis, 2007), and changes in parental investment are typically considered in terms of new vs. experienced parents, or in other words primiparous vs. multiparous parents. Some studies report that multiparous female rats and mice (notably, both generally monoparental species), when rearing pups alone, are more responsive to pups than primiparous conspecifics (Kinsley, 1994), while others report no difference (McGuire and Bemis, 2007). However, when presented with a sexually mature male conspecific, multiparous female Norway rats in post-partum estrus more effectively switch between maternal and copulatory behaviors than primiparous mothers, as in part indicated by more effective pup retrievals (Gilbert et al., 1984). Previous study of the biparental prairie vole is conflicted, either demonstrating that multiparous females display more parental care than primiparous females (Wang and Novak, 1994a) or that primiparous females display more parental care than multiparous females (Stone and Bales, 2010). Paternal care either does not change (Wang and Novak, 1994a) or is greater in primiparous than multiparous prairie vole fathers (Stone and Bales, 2010). In the biparental coppery titi monkey, infants reared by multiparous mothers are more likely to survive than those reared by primiparous mothers, although neither infant growth rate nor maternal behavior are shown to be different between primiparous and multiparous mothers (Jarcho et al., 2012). In humans, first-time parents of both sexes show more arousal to cues of infant crying than non-parents and experienced parents (Boukydis and Burgess, 1982).

Notably, previous study of the effect of parity on parental behavior has been primarily conducted in mothers, with little research on the effect of parity on paternal care or on the interplay between maternal and paternal care. A synthesis of the findings in biparental species would indicate that parity has no effect on parental care (Jarcho et al., 2012), that more parental experience begets more parental attention and care (Boukydis and Burgess, 1982; Wang and Novak, 1994a), or that parental experience yields a decrease in parental care (Stone and Bales, 2010). Here, we briefly review evidence from one model monogamous and biparental species, the prairie vole (*Microtus ochrogaster*), suggesting an important role of paternal care in the development of the monogamous behavioral phenotype. We then consider the connection between parental experience and parental behavior using a novel application of latent growth curve modeling.

The prairie vole is a socially monogamous arvicoline rodent native to the North American Midwest (for review on taxonomy, conservation, and distribution, see Laerm and Ford, 2007). Field observations of prairie voles are relatively difficult, as the species navigates their environment along running paths in tall grass and nests in underground burrows (Gier and Cooksey, 1967). The dominant mating system among prairie voles is monogamy, with family units consisting of the breeding female and male along with their offspring, of which the majority will remain in their natal nest living with parents and other siblings for life (Getz and Carter, 1980; Getz et al., 1981, 1987, 2003, 2005; McGuire et al., 1993; McGuire and Getz, 2012). Under natural conditions, the average tenure of a breeding pair is short (approximately 42 ± 14 days) with termination most often due to the death of one or both partners (Getz and Hofmann, 1986); it is unlikely that new pair bonds are formed following the loss of a mate (Carter et al., 1997; Pizzuto and Getz, 1998).

More direct observation of mating and parenting behaviors was facilitated by the successful integration of prairie voles into the laboratory in the late 1960s (Gier and Cooksey, 1967). Ovulation in female prairie voles is induced in the presence of male prairie voles (Carter et al., 1980), and both sexes demonstrate incest avoidance (McGuire and Getz, 1981). Gestation for the species is 20–21 days, and successive litters of pups may be born every 21–24 days (Gier and Cooksey, 1967). Prairie vole mothers and fathers both demonstrate a high frequency of parental behavior in laboratory and in seminatural conditions (Hartung and Dewsbury, 1979; Thomas and Birney, 1979; Solomon, 1993; Lonstein and de Vries, 1999; McGuire et al., 2007). The pattern of prairie vole parental care is sexually dimorphic, with mothers investing more time in direct pup-directed behaviors and nest maintenance than fathers (Solomon, 1993; McGuire et al., 2007; although, see Hartung and Dewsbury, 1979).

Biparental care in prairie voles persists throughout the pre-weaning period (post-natal days 0–21), and maternal behavior (e.g., brooding, nursing, and grooming) and time in nest is greatest in the perinatal period and declines across the pre-weaning period (McGuire and Novak, 1984). Natural variation in frequency and type of prairie vole parenting behavior in the perinatal period has been demonstrated to have significant consequences for bio-behavioral development of offspring (Perkeybile et al., 2013; Arias Del Razo and Bales, 2016). Directly observable developmental consequences result from natural variation in the quantity of parental care given by prairie vole parents to their offspring (i.e., pups)–that is, pups reared by low contact parents develop more quickly than conspecifics reared by high contact parents, as demonstrated through the advent of developmental milestones (i.e., opening of eyes, eating solid food, leaving the nest autonomously, etc.; Perkeybile et al., 2013; although, see Wang and Novak, 1994b). Increased parental contact promotes social affiliation (Arias Del Razo and Bales, 2016), and the behavioral phenotypes of monogamy (e.g., partner preference formation) are also facilitated by increased parental contact (Bales et al., 2007; Ahern and Young, 2009; Stone and Bales, 2010). Natural variation in prairie vole parental behavior induces differences in the size of pup cortical fields (Seelke et al., 2016a), intrinsic connections within the primary somatosensory cortex (Seelke et al., 2016b), as well as neuroendocrine function (Perkeybile and Bales, 2016). For a review of possible mechanisms behind these effects, see Perkeybile and Bales (2017).

While the developmental consequences of variation in parental care are becoming increasingly understood, the dynamic processes that result in observable patterns of parental behavior remain understudied. One might hypothesize that, in rodents, parental behavior remains consistent across litters, that is, parental behavior does not significantly vary from one litter to the next. One might also hypothesize that parental behavior in a monogamous, biparental species ought to be characterized as a dyadic process with extensive coordination and mutual-reliance between partner parents. Under seminatural conditions, pups reared under biparental care are left unattended less often than conspecifics reared under mother only conditions, suggesting some level of coordination between mothers and fathers (McGuire et al., 2007); however, previous study of maternal care under conditions of paternal absence also presents little evidence for maternal compensation for paternal loss, suggesting that parenting in prairie vole mothers may function independently of the paternal behavior of their partner (McGuire et al., 2007; Ahern et al., 2011). Still, there remains little empirical evidence to either support or reject these conceptualizations of prairie vole parenting.

In this study, we seek to explore two behavioral processes with the potential to alter developmental trajectories of monogamy in offspring: the nature of parenting across litters; and, the nature of parenting between partner parents. In the case of the former, we consider if parental care changes from the first litter and across a series of subsequent litters, or if parental care does in fact remain consistent across litters. For the latter process, we consider the extent to which individual parents’ behaviors are influenced by those behaviors of their partner. Here, we hypothesize consistency of parental care across litters, and we hypothesize that parenting behavior in prairie voles can be characterized as a coordinated process.

Latent Growth Curve Models (LGMs) are a useful way to conceptualize and analyze hypothesized processes of change, including change in means (McArdle and Epstein, 1987). LGMs can be applied, even using incomplete data, for analysis of processes that display no growth (level-only) and/or linear or non-linear models that include both an intercept and slope (Ferrer et al., 2004). These models can incorporate covariates (both time-varying and time-invariant) to examine the effect of external factors on the process (McArdle and Epstein, 1987). Ultimately, these models can also be expanded to accommodate multivariate processes (McArdle, 1988, 2009; Ferrer and McArdle, 2003; Bollen and Curran, 2006).

Here, we attempt to use all of these methods to better characterize the nature of prairie vole parenting as it unfolds across a pair’s first 4 l. We consider models of no growth and change at the level of the mother, father, and dyad; and we attempt integration of covariates of parental age and litter size. Ultimately, such a characterization is needed to ascertain what potential there might be for parental care to directly influence behavioral phenotypes of monogamy (e.g., partner preference, selective aggression, etc.) in offspring. Because offspring phenotypes of monogamy are directly influenced by parental care, characterization of change in parental care elucidates our understanding of how phenotypes of monogamy may or may not vary from one litter to the next. This study explores the stability of parental care, a moderator of monogamous phenotypes, to better inform our understanding of prairie vole parenting as a process.

The use of LGMs to analyze longitudinal trajectories of biparental care in prairie voles is, to the best of our knowledge, novel. Estimates of trajectories of biparental care produced using LGMs take into account within-parent trajectories across subsequent litters, which should yield a more reliable prediction of a trajectory generalizable across all observed individuals (Bollen and Curran, 2006). This approach contrasts with cross-sectional methods used in previous studies on the effect of parity on prairie vole parental care. Moreover, where previous studies have compared primiparous and multiparous mothers, our study uses a litter-to-litter approach to capture a more detailed, dynamic process. Finally, this study also attempts to bring both maternal and paternal care into the context of the biparental dyad, i.e., how maternal and paternal care compare and contrast across subsequent litters.

## METHODS

### Research Questions

Here, the two behavioral processes we sought to investigate were explored in a series of questions. A fundamental question concerned the true unit of analysis—that is, should the level of analysis be *the individual* or *the dyad*? We first considered if, when treated as independent actors, male and female parents demonstrate unique patterns of change across four sequentially reared litters. Further, we considered if change can be best characterized with a model of no growth, linear growth, or non-linear growth. A model of no growth could be indicative of either constant (e.g., invariant) parenting or an inability to identify a mean trajectory followed by all parents. Support for a linear growth model would indicate an approximately equal growth or decline of the mean parental behavior across litters. A non-linear growth model would indicate growth or decline of the mean parental behavior in an unequal way across litters. We made the same considerations of change at the level of the dyad, using a model that considers the dyad as two covarying individuals— that is, we considered an alternative model in which the level of analysis is the individual (either male or female parent), but in which some interaction between partner parents is permitted through time-varying covariances.

### Subjects and Laboratory Conditions

All data were collected from the prairie vole colony at the University of California, Davis over a period of approximately 6 years. One-hundred forty-one parenting dyads were selected from archival data on the basis that no experimental manipulation had been applied to the dyad or their pups and that observations of parenting behavior had been made upon the dyad during post-natal day (PND) one through three. All subjects were descendants of wild prairie voles that were originally caught near Champaign, Illinois and continually outbred to maintain genetic diversity, and included new genetic stock from another captive colony in 2015. Animals were maintained on a 14:10 light-dark cycle with lights on at 06:00. Water and food (high-fiber Purina rabbit chow) were provided *ad libitum.* Breeder pairs and their offspring were maintained and observed in large, polycarbonate cages (44 × 22 × 16 cm). Each cage had aspen wood bedding (i.e., Sani-Chips), and cotton was provided for nesting material. Humidity was controlled and room temperature maintained near 70^◦^F. All pups were weaned from the home cage on PND20, and subsequent litters of pups were not exposed to older siblings. Home cages were left undisturbed beyond weekly cage changes and three daily checks of food and water.

Litter size for each cage was recorded at PND1 and corrected at weaning in the event of a miscount. Corresponding information on litter size in each observed litter and the age of each parent at the time of birth of their first litter was also found in laboratory archives and recorded when available. The mean number of pups per litter across all measured litters (1–4) was 4.75 (*SD* = 1.47); the number of pups per litter ranged from 1 to 8. At the time of birth of their first litter, mothers’ mean age was 99.56 days (*SD* = 26.56); the range of maternal age at first litter was 64 to 180 days. At the same time point, the fathers had a mean age of 145 days (*SD* = 73.00); the range of paternal age at first litter was 51–422 days.

When measures of parental behavior were (in some cases) available for many litters, litters one through four were used in this study. This decision was both practical and theoretical. Practically, the availability of observational data beyond litter four was increasingly sparse. Under natural conditions, prairie vole dyads are unlikely to produce more than four litters of pups before the death of one or both parents, as the average life expectancy of both male and female prairie voles is short (approximately 50–80 days), with most breeding pairs surviving only 1–2 months (Getz and Hofmann, 1986; Getz et al., 1997, 2000; Thomas and Wolff, 2004). In laboratory conditions, animals may live over 2 years and breeding pairs may produce well in excess of 10 successive litters.

### Behavioral Measures

Parental care observations were collected through live focal sampling by graduate students and undergraduate research assistants who were trained and validated according to the ethogram presented in Perkeybile et al. (2013). Observers sat approximately 1–2 feet from the home cage, and observations were recorded on laptop computers using behavioral software (www.behaviortracker.com). For each litter, both mother and father were simultaneously observed for 20 min focal samples during PND1–3. Mothers were distinguished from fathers using individual characteristics (e.g., size, color, etc.) in addition to observations of pup attachment to nipples or evidence that nursing had occurred (e.g., swollen nipples or milk-wetted fur). Variation in parental behavior in this perinatal period has been predictive of pup bio-behavioral outcomes (Perkeybile et al., 2013). The perinatal period is also the period in which the most extensive parental care is shown, and it is a period in which, prior to fur growth, pups have little ability to thermoregulate (Gebczynski, 1981; Blake, 1992). The observed home cage was left undisturbed on a metal cage rack with overhead coverage from the rack above. Observations were not conducted on days on which cage changes occurred.

The primary outcome measure was a composite score of pup-directed behaviors (PDBs), which was calculated for each respective litter from the focal sampling output. The number of seconds spent in a variety of pup-directed behaviors (i.e., nursing, licking and grooming, physical contact, etc.) were summed across each focal sample, added across all focal samples for each respective litter, and then divided by the total number of focal samples collected (between two and four) for a mean value of pup-directed behavior observed for each parent in the perinatal period of each respective litter. In all, 93.2% of composite scores were generated from four focal samples, 4.1% from three focal samples, and 2.6% from two focal samples with a mean of 3.91 (median = 4) focal samples per composite score of PDBs. Some behaviors were not considered mutually exclusive (e.g., nursing and licking), thus allowing for summed totals of PDB to exceed 1,200 s; and maternal and paternal care were often contemporaneous.

### Missing Data

The mean number of litters observed per breeder pair was 2.13 (median = 2 litters). Across all 141 breeding pairs and across all four time points, 303 litters were observed; Thus, of the 564 potential opportunities for observation (i.e., across all 141 breeder pairs at four time points), there were 261 missing observations, due to attrition across the 4 litters. A complete summary of the distribution of missing observations (by litter) is provided in Table 1. The number of missing observations in the data increased as the litter number increased from 19 in litter 1 to 108 in litter 4. Data were not missing because of active researcher selection for any particular behavioral trait(s). This behavioral paradigm was designed originally as a behavioral diagnostic to classify new parents as low-, medium, or high-contact parents, and the diagnostic was originally designed such that generally only the first two litters of a pair were observed. However, in some circumstances, later litters (i.e., litters 3 and 4) had been opportunistically observed. The choice to halt observation after two litters or to continue observation in later litters was not determined by any behavioral characteristic of the pairs observed. For some pairs, these observations were not necessarily in consecutive litters (due to inconvenience, logistic difficulty, etc. of observation timing). For example, for some pairs there are observations for litters 1 and 3, rather than litters 1 and 2; and, for others there were observations for litters 1–4. Thus, we believe it is reasonable to assume data to be missing completely at random (MCAR), and we do not expect missing observations to introduce bias into the estimated model parameters.

### Model Specification

In this study, we explored three separate clusters of models. The first cluster contains two identical sets of models for mothers and fathers. The second cluster adds to the cluster 1 models both a time-varying covariate for age and a time-varying covariate for litter size. The third cluster allows for time-varying covariance between parenting partners—that is, if a significant covariation exists between maternal and paternal care, and how that covariation exists across successive litters. The level of analysis was primarily exploratory, with the primary interest being in model selection based on comparative indices of model fit, i.e., what pattern of change best describes the observed data on parental care across successive litters. Within each cluster, models of *no growth*, *linear growth*, and *non-linear growth* were compared in a structural equation modeling framework (see Kline, 2016 for further reading) to determine which growth pattern best fit observed data from mothers and fathers independently (i.e., independent maternal and paternal models), and mothers and fathers within the context of one-another (i.e., a maternal-paternal bivariate model). The models run for mothers and fathers could not be compared directly to each other given different data were used for the generation of their respective models. However, model parameters for each sample can be interpreted and compared (yet informally) across samples. Path diagrams representing the respective models are found in (Figures 3A–C) along with detail for their interpretation. For further reading on these techniques, we recommend Bollen and Curran (2006).

#### Model Cluster 1: Independent Maternal and Paternal Models

**FIGURE 1 |:**
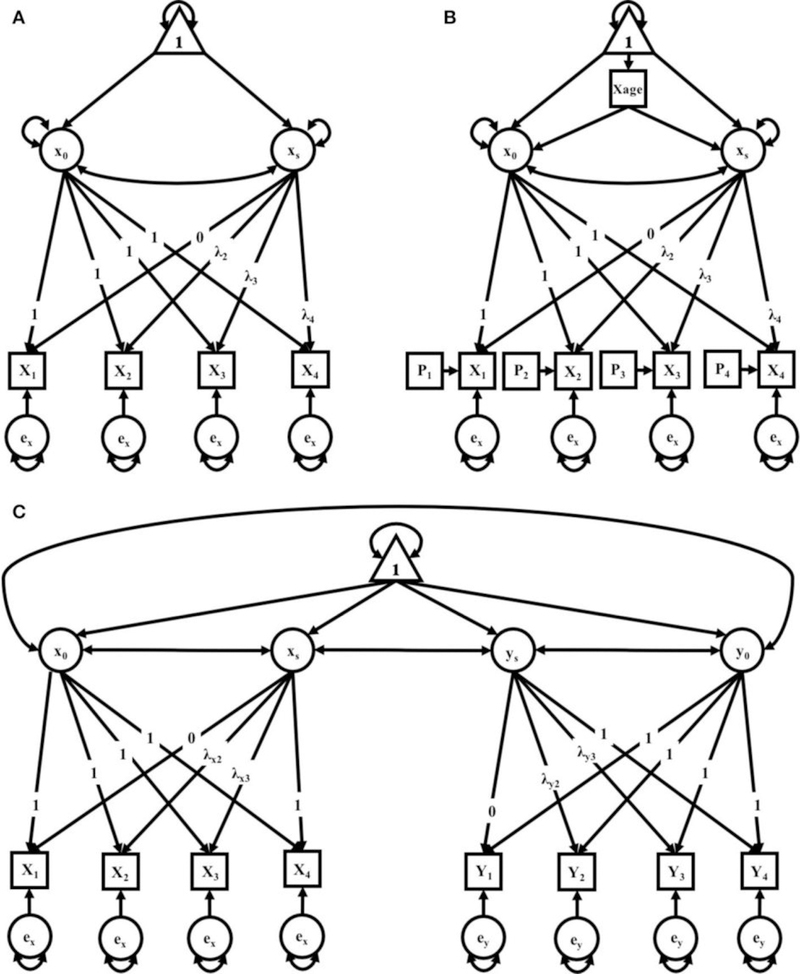
**(A–C)** path diagrams for Clusters 1–3. Path diagrams are visual representations of model parameters and their relationships, in which each type of model parameter is provided a graphical symbol, i.e., squares or rectangles are used for observed variables (i.e., indicators), circles are used for latent variables and error terms, straight arrows for hypothetical causal or direct effects, and curved arrows for covariances (Kline, 2016). In all path diagrams presented here **(A–C)**, composite scores of pub-directed behaviors (PDBs) for each litter (1–4) are represented as sequential squares (from left to right) around indicators by X_t_ or Y_t_, where t = litter. Each represented composite score (e.g., X_t_ or Y_t_) has an error term, e_x_ or e_y_, which represents variance not explained by the composite scores, including variance due to measurement error. Elements of regression, intercept and slope, are represented by x_0_ and x_s_, respectively, and circumscribed to represent their status as latent variables, each with their own covariance and a covariance between the two. Triangles containing the number 1 represent the inclusion of a term for the analysis of means. As the intercept for each model does not change across time, straight arrows from the intercept factor to each time point are labeled with the number 1, indicating a constraint on estimation of the regression between the intercept and each time point. Straight arrows from the slope factor (x_s_ or y_s_) are variably constrained, according to the growth pattern in consideration. Thus, for the no growth model, regression paths from the slope factor to indicators at all time points are constrained to 0; for the linear growth model, regression paths from the slope factor to indicators are constrained to 0, .33, .67, and 1 for litters 1, 2, 3, and 4, respectively; and for the non-linear growth model, only the regression path from the slope factor to litter 1 is constrained (to 0) and all others are freely estimated, as represented by the term λ_*t*_. Path diagram **(A)** represents models tested in Cluster 1, the Independent Maternal and Paternal Models, which includes only the common parameters outlined above. Path diagram **(B)** represents models tested in Cluster 2, the Independent Maternal and Paternal Models with Covariates, which expands upon the model given for Cluster 1 with the addition of a time-invariant covariate for parental age (X_age_) and time-varying covariates for litter size (P_t_), both given in squares. Path diagram **(C)** represents models tested in Cluster 3, the Maternal-Paternal Bivariate Model, which expands upon the model given for Cluster 1 by presenting models for change in maternal and paternal care in parallel with double headed arrows representing covariance between the two models, and therefore covarying trajectories of change between mothers and fathers.

There were three models in this cluster: a No-Growth Model, a Linear Growth Model, and a Non-linear Growth Model (see Figure 1A). All three models include an observed outcome measure of a composite score of pup-directed behaviors for each litter (1–4).

The No-Growth Model for the maternal parent is therefore expressed as:

$$X_{\left[t\right]n}=x_{\left[0\right]n}+e_{\left[t\right]n}\;x_{\left[0\right]n}=\mu_{x\left[0\right]}+d_{x\left[0\right]n}$$

where *X*_[t]*n*_ is the observed score for the outcome variable (i.e., PDBs) at any given point, t (i.e., litter 1–4), x_[0]*n*_ is an individual’s intercept (i.e., PDB for litter 1), and e_[t]*n*_ is the residual variance; µ is a fixed group mean, and *d* represents the variation in such mean across individuals. Whereas the no-growth model predicts only a starting value (i.e., the intercept) and no further change, this model is expanded upon in the Linear Growth Model and the Non-linear Growth Model to include an individual’s slope (i.e., pattern of change in PDBs from litter 1 onward). Thus, the general model is:

$$X_{\left[t\right]n}=x_{\left[0\right]n}+B_{\left[t\right]}\cdot x_{sn}+e_{\left[t\right]n}\;x_{\left[0\right]n}=\mu_{x\left[0\right]}+d_{x\left[0\right]n}\;x_{\left[s\right]n}=\mu_{x\left[s\right]}+d_{x\left[s\right]n}$$

where *B*_[*t*]_ represents a set of coefficients expressing the shape of the curve. These coefficients can be fixed to test various hypotheses of growth. For example, for a linear growth model, the coefficients can be fixed as = 0, 0.33, 0.67, 1, such that the effect of parity on the outcome of PDBs is constrained to a linear pattern. Alternatively, some of the *B*_[*t*]_ can be estimated from the data to detect non-linear changes, thus to test the Non-linear Growth Model, which is also often referred to as a latent basis model. For this, the first and last loading (leading from x_s_ to the PDBs for litters 1 and 4, respectively) can be set to 0 and 1, respectively, and the loadings for litters 2 and 3 are left to be freely estimated from the data.

#### Model Cluster 2: Independent Maternal and Paternal Models With Covariates

Cluster two expanded on the models in cluster one with the addition of covariates for parental age at the birth of their first litter and litter size for each litter (see Figure 1B). There were similarly three models in this cluster: a No-Growth Model, a Linear Model, and a Non-linear Growth Model. All models included an observed measure for each litter (1–4), each with its own residual and time-varying covariate (*P*_*t*_ ) to account for litter size. Both models also accounted for parental age with the inclusion of an exogenous, time-invariant covariate for age (Xage, Yage, respectively). In the models generated for the combined dyad, both exogenous covariates for age are included. The model for which time does not contribute to a pattern of growth for the maternal parent is expressed as:

$$X_{\left[t\right]n}=x_{\left[0\right]n}+p_{\left[t\right]n}+Xage_{n}+e_{\left[t\right]n}\;x_{\left[0\right]n}=\mu_{x\left[0\right]}+d_{x\left[0\right]n}$$

#### Model Cluster 3: Maternal-Paternal Bivariate Model

Cluster 3 took the same elements from the first cluster to form a bivariate growth model, which estimates two individual models (maternal and paternal) simultaneously. This third cluster of models allows for covariation between the female and male parent such that the intercepts and slopes of the two covary. Here, again, factor loadings could be fixed to = 0, .33, .67, 1, or left open and allowed to vary freely in order to form the Non-linear Growth Model (see Figure 1C); specifically, maternal factor loadings were fixed for a linear model while paternal factor loadings were fixed for a non-linear growth model, according to the best fitting models in Cluster 1. Either could be modified, as in cluster 2, to include either or both time-invariant and/or time-variant covariates. This model affords new parameter estimates, including the covariance between slopes of each parent (i.e., the extent to which changes are related), as well as the covariance between the parents’ intercepts.

### Statistical Analyses

All models were fit with the package *lavaan* (Rosseel, 2012) in R (R Core Team, 2017) using full information maximum likelihood (FIML). All behavioral scores were divided by 100 to put all measures in the same metric and aid convergence. Standard measures of model fit (chi-square difference test, CFI, TLI, RMSEA) and information criteria [AIC_i_, Δ_i_(AIC), and w_i_(AIC)] were used to evaluate the models.

“Good fit” is first characterized, here, by a *p*-value above 0.05, an indication that the hypothesis of exact model fit is not rejected. Ideal Comparative Fit Index (CFI; Bentler, 1990) and Tucker-Lewis Index (TLI; Tucker and Lewis, 1973) scores are >0.95 (Hu and Bentler, 1999). Better Root Mean Square Error of Approximation (RMSEA; Steiger, 1990) scores are below 0.080. A chi-square difference test can be used to determine significant differences (improvement) in fit between two models that are nested, that is, when one model can be represented as a more constrained sub-model of the other. Thus, our no growth model is nested within linear growth model, which is in turn nested within the non-linear growth model.

The additional measure of fit, Akaike’s Information Criterion (AIC_i_), was generated as well as transformations of AIC_i_, including 1_i_(AIC), the change in AIC between each model and the best candidate model, and Akaike Weights [w_i_(AIC)], which can be interpreted as a probability that a model is the best in its set (Wagenmakers and Farrell, 2004). All fit indices for Cluster 1 and Cluster 3 can be found in Table 3.

In some cases, there was an occurrence of a *Heywood Case*, an instance in which there are parameter estimates of illogical value (Chen et al., 2001; Kolenikov and Bollen, 2012). Here, such cases were restricted to the production of negative slope variances; thus, where the model with negative variances demonstrated significant improvement of model fit over alternative models, the variance and all covariances for the slope parameter were fixed to zero and treated as a “corrected” model. Corrected latent base models are comparable to no-growth models, as the no-growth models are nested within the latent base model; model comparison between corrected latent base models and linear models is, however, impossible for lack of nesting.

## RESULTS

### Descriptive Statistics

**FIGURE 2 |:**
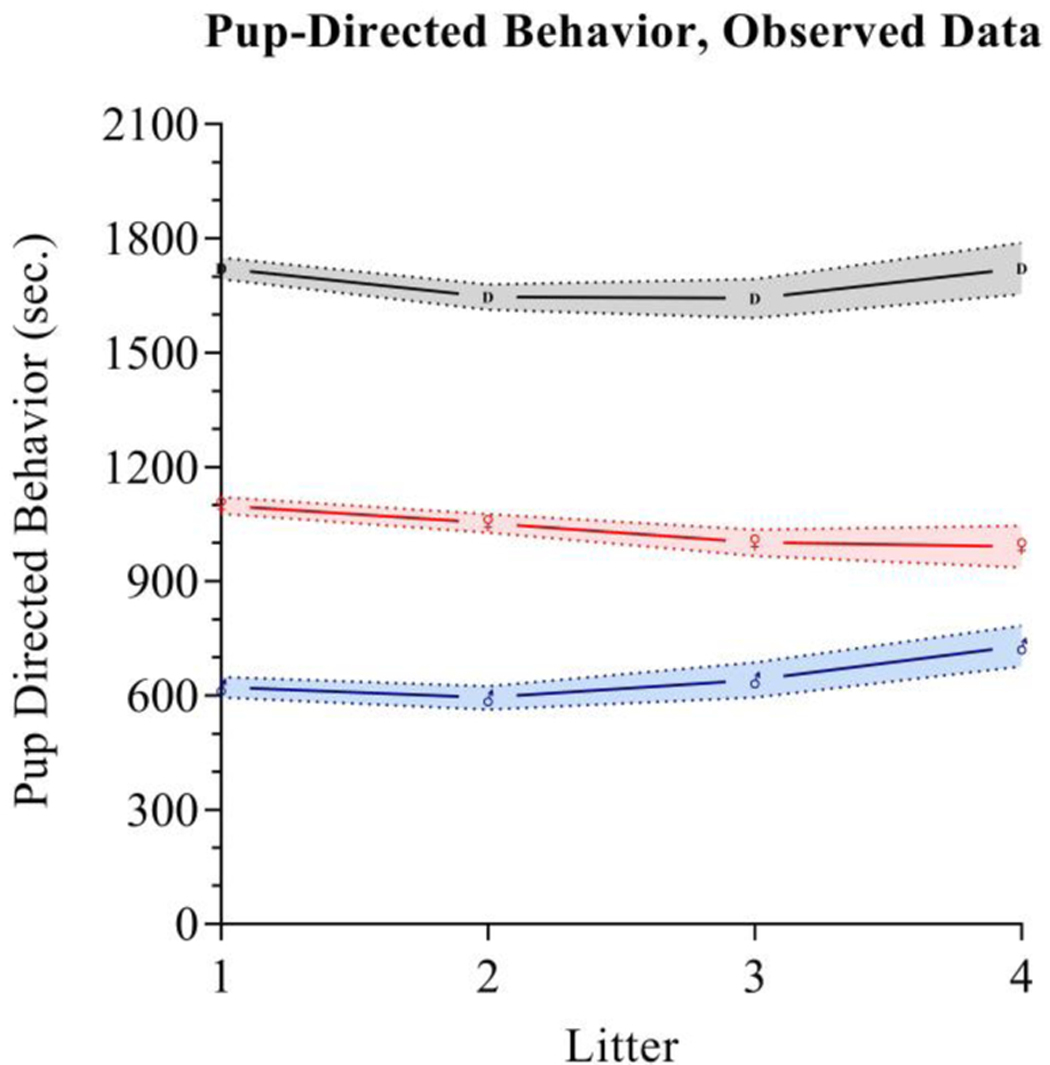
Parental care observed data. Pup directed behaviors are tracked for fathers (blue), mothers (red), and as a dyadic composite (black) across the first four litters. Mean values for each group at each time are respectively indicated by the Mars symbol (♂), Venus symbol (♀), or a “D”. The shading in blue, red, and gray respectively indicates standard error intervals around the means.

Maternal pup-directed behaviors have a mean value of 1,055 (*SD* = 251.8) with a range of 250–1,522. Maternal PDBs appear close to normally distributed with a slight negative skew and kurtosis. Paternal PDBs have a mean value of 628.5 (*SD* = 307.9) with a range of 0–1,366. Paternal PDBs appear to be normally distributed with positive skewness and kurtosis. Combined dyad PDBs (i.e., the sum of maternal and paternal PDBs) have a mean value of 1,684 (*SD* = 335.9) with a range of 585.8–2606.0. Dyad PDBs are normally distributed. The slope of plotted observed means across the four measured litters for maternal PDB, paternal PDB, and combined dyad PDB appear flat-to-negative, flat-to-positive, and flat-to-positive, respectively (see Figure 2). Correlation coefficients between maternal and paternal care care at each time point are presented in Table 2.

### Independent Maternal and Paternal Models

**FIGURE 3 |:**
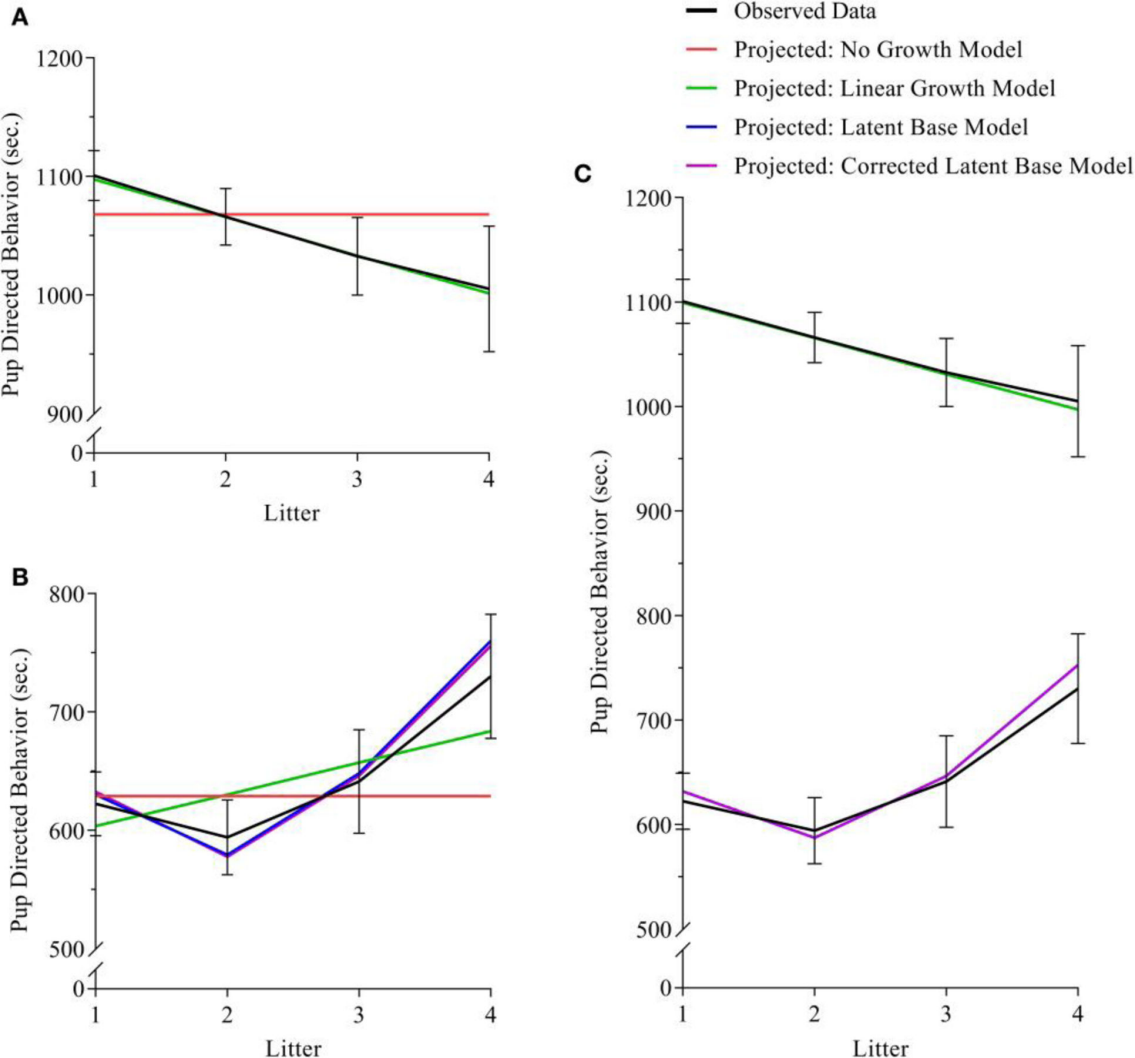
**(A–C) (A)** maternal, **(B)** paternal, and **(C)** bivariate dyadic models of pup-directed behavior. In all sub-figures: black lines indicate the plotted means of the sample data; red lines indicated the plotted means of the calculated no-growth model; green lines indicate the plotted means of the calculated linear model; blue and purple lines indicate the plotted means of the uncorrected and corrected latent base models, respectively. In Figure 1C, the black line in the lower half of the figure indicates the means of sample data for fathers, and the black line in the upper half of the figure indicates the means of sample data for mothers. Black error bars represent standard error.

For analyses of maternal care, the exact-fit hypothesis was not rejected for no-growth $\left[\chi_{M}^{2}\left(11\right)=18.521,p=0.070\right]$, linear $\left[\chi_{M}^{2}\left(8\right)=9.923,p=0.318\right]$, and latent base $\left[\chi_{M}^{2}\left(6\right)=10.194,p=0.117\right]$ models, giving first evidence that all three models could fit the observed data. A chi-square difference test shows that the addition of a linear growth parameter significantly improves model fit compared to the no-growth model $\left[\chi_{D}^{2}\left(3\right)=9.229,p=0.026\right]$ (see Table 3, Figure 3A). The non-linear growth model, however, does not significantly improve model fit over the no-growth model $\left[\chi_{D}^{2}\left(5\right)=8.328,p=0.139\right]$ CFI, TLI, RMSEA, and AIC_i_ show better fit for the linear model over the no-growth model, and w_i_(AIC) sets the conditional probability that the linear model is the best fitting model of the set at 0.778.

For analyses of paternal care, the exact-fit hypothesis is not rejected for no-growth $\left[\chi_{M}^{2}\left(11\right)=17.246,p=0.101\right]$, linear $[\chi_{M}^{2}\left(8\right)=15.021,p=0.059,$ and latent base $\left[\chi_{M}^{2}\left(6\right)=6.537,p=0.366\right]$ models, giving first evidence that all three models could fit the observed data; however, the non-linear growth model demonstrated negative variance while also displaying better relative fit (as assessed by CFI, TLI, RMSEA, and AIC_i_) than either the no-growth and linear models. For a corrected non-linear growth model, the exact-fit hypothesis is also not rejected $\left[\chi_{M}^{2}\left(8\right)=5.565,p=0.585\right]$ and the corrected latent base model shows improvement of model fit compared to the no-growth model $\left[\chi_{D}^{2}\left(3\right)=10.681,p=0.014\right]$ (see Table 3, Figure 3B). The conditional probability that the corrected non-linear growth model is the best fitting model of the set is 0.912.

### Independent Maternal and Paternal Models With Covariates

For both instances (maternal care and paternal care), the addition of covariates controlling for age and litter size yielded models with exceptionally poor fit. Cluster 2 models yield estimates of intercept and slope that are comparable, yet further from the observed data, to those from models in Cluster 1.

### Maternal-Paternal Bivariate Model

For analyses of parental care in a corrected bivariate process, in which the trajectory for maternal care is fixed as linear and the trajectory for paternal care is fixed as non-linear, the exact-fit hypothesis is not rejected $\left[\chi_{M}^{2}\;(24)\;=\;30.616,\;p\;=\;0.165\right]$. The result of negative slope variance for paternal care and subsequent correction for the Heywood Case means results for slope covariance between maternal and paternal care are uninterpretable. The covariance of intercepts for maternal and paternal care is −0.057 (*p* = 0.93) (see Table 3, Figure 3C).

## DISCUSSION

### Parenting Changes Over Time

We hypothesized that prairie vole parental care does not vary from one litter to the next. Our results depart from this hypothesis of consistency. Contrary to previous observations that indicated increased parental care in multiparous mothers (Wang and Novak, 1994b), our findings suggest that maternal care is best described as having a negative trajectory, with a decline across litters (see Figures 3A,C). Differences in methodology may explain these differences, particularly the use of cross-sectional vs. mixed-longitudinal analysis in the former and current studies, respectively, as well as significant differences in sample size. Previous study of parental change and parity in male and female prairie vole parents from litter 1 and 2 (using longitudinal methods) also find a decline in parental care from litters 1 and 2 (Stone and Bales, 2010). Moreover, our findings of decline in maternal care with parental experience aligns more closely with the human literature (Boukydis and Burgess, 1982). The mechanism behind a decline in care for neonates across subsequent litters is not particularly clear. We hypothesize that mothers may become more efficient in their investment with parenting experience; alternatively, reduced sensitivity to pup cues may play some role, as could the effect of aging. *Post-hoc* investigation of the individual behaviors that compose the composite score for PDBs show that decline in maternal care is reflected in time nursing (Supplementary Table 1, Supplementary Figure 3); a similar decline is observed in time spent in the nest (Supplementary Table 1, Supplementary Figure 1), which is not a component of the composite score for PDBs.

Paternal care is best described as having an initial decline from the first to second litter and a subsequent uptake in paternal activity from litter 2 through 4 (see Figures 3B,C). The comparison of mothers’ and fathers’ respective patterns of parental care highlights their opposing trajectories (Figures 2, 3C). A pattern of initial decline followed by an increase in paternal behavior could be explained by competing factors; that is, one responsible for the decline, and another which counteracts the decline. Similar mechanisms behind maternal care decline could be responsible for paternal care decline, including an improved paternal efficiency, reduced sensitivity, or a broad effect of aging. We would then hypothesize that paternal care increases following an initial decline in order to compensate for maternal decline in investment in of their offspring. *Post-hoc* investigation of paternal care shows that the paternal pattern of decline and subsequent incline is reflected in paternal time in nest (Supplementary Table 1, Supplementary Figure 2), which is not a component of the composite score for PDBs. The increase of PDBs appears to be driven largely by an increase in huddling and non-huddling contact (Supplementary Table 1, Supplementary Figures 5, 7, respectively). Other individual parental behaviors are displayed in Supplementary Figures 4, 6, 8–11.

### Compensatory Fathers

The models of individual change for mothers and fathers fit quite well and suggest opposing trends in parental care between mothers and fathers from litters 2 to 4. The general stability seen in the composite of maternal and paternal behavior (Figure 2) may be a consequence of opposing trends, where when mothers decline in maternal care across time, fathers display compensatory behavior to fill an apparent void. We hypothesize that the increase of paternal care could be a response intended to decrease the amount of time pups are left unattended on the nest, or more simply that fathers are aware of unattended pups and act to ameliorate the pups’ condition. A potential mechanism by which fathers become aware of unattended pups is through pup ultrasonic vocalization, to which male prairie voles are responsive (Rabon et al., 2001). The mechanism behind paternal motivation to respond to ultrasonic vocalization may be mediated through stress, as pup-vocalizations may be stress inducing and male prairie voles have been demonstrated to increase parental care when stressed (Bales et al., 2006). Moreover, the initial decline and subsequent increase of paternal care parallels previous trends of weight loss and subsequent recovery in fathers across their first 3 l of pups (Campbell et al., 2009). This suggests energetics may play an important role in the patterns of paternal behavior.

One challenge to the hypothesis of independent actors is the observation of “forced baby sitting”—an behavior in which mothers or fathers may push one another back into the nest—, which, while anecdotal, we and others have observed in prairie voles under laboratory conditions (although, not seen in McGuire et al., 2007) and empirically observed in other vole species (e.g., *M. socialis guentheri*; Libhaber and Eilam, 2002); thus, it is possible that mothers increasingly encourage paternal behavior through forced baby sitting with each successive litter.

### Consequences for Offspring

Responsive fathers may play an important role in stabilizing early life experiences for offspring, particularly offspring of later litters which experience less maternal care. Without the stabilizing influence of fathers, pups in later litters might experience significantly less direct parental care than those of earlier litters. Despite shared genetics, we could then expect differential adult behavioral phenotypes in siblings of different litters if fathers cannot or do not stabilize the parental environment.

In prairie voles, increased parental investment may result in higher survival rate in the laboratory (Wang and Novak, 1994a), although other studies would suggest no such effect in the laboratory setting (Mcguire et al., 1992; McGuire et al., 2007; Wang and Novak, 1992). Under more challenging circumstances, the conferred benefit of paternal care on pup survival may be more pronounced in some biparental species, such as under conditions of cold temperature (in California mice, Gubernick et al., 1993), presence of predators (in prairie voles, Getz et al., 1992), or increased effort to access food (in California mice, Gubernick et al., 1993; Cantoni and Brown, 1997). High contact parenting is also associated with slower development, as indicated through the achievement of developmental milestones of eye opening, leaving the nest, and eating solid food (Perkeybile et al., 2013). Contrasts between high- and low-contact parents also indicate variation in neuroendocrine (Perkeybile and Bales, 2016) and cortical development (Seelke et al., 2016a); for example, pups receiving high tactile contact develop greater intrinsic and more localized connections in the primary somatosensory area than their low-contact counterparts (Seelke et al., 2016b).

Of particular relevance here is the expression of monogamy in adulthood, which has been linked to early life experience of parental care. Experimental manipulations of the family unit during the neonatal period induce increased parental behavior, an effect that resulted in facilitated partner preference formation in manipulated (higher contact) female offspring, but inhibited partner preference formation in non-manipulated (lower-contact) female offspring (Bales et al., 2007). High contact parenting as a result of natural variation also promotes social behavior (e.g., physical contact) when compared to medium-and low-contact parenting (Arias Del Razo and Bales, 2016). These effects are demonstrated to have intergenerational effects on parenting and alloparenting behaviors (Stone and Bales, 2010; Arias Del Razo and Bales, 2016). After paternal deprivation, male and female offspring reared with their mother only show deficits in pair-bond formation, thus indicating that paternal care facilitates partner preference formation (Ahern and Young, 2009).

Another potential stabilizing factor not considered here is the presence of alloparental care from non-dispersed siblings. The presence of fathers and alloparental siblings is associated with decline in maternal care and time spent in the natal nest as well as increased maternal locomotion, eating, and drinking (Wang and Novak, 1992). Pups reared in the presence of a mother, father and alloparents are left unattended less frequently than those reared with only a mother (Solomon, 1991). While well reviewed by Kenkel et al. (2017), more research on the effects of alloparental care on early life development is needed.

### Limitations and Future Directions

A major limitation of this study is the relatively small sample size of 141 dyads (282 individuals), which is compounded by a high rate of missing data. As discussed earlier, the missing data are likely to be missing completely at random, which means that they have little potential to introduce systematic bias into the results. However, missing data translate directly into loss of power and loss of precision in the estimation. Continued data collection, particularly in later litters (e.g., litters 3 and 4) would be particularly beneficial to future analyses. Another benefit of an increased sample size would be the ability to utilize multiple groups models to consider the classification of “high contact” and “low contact” parents—that is, parents who show stability in the extremes of parenting over time. It should also be noted that our analyses do not provide information as to whether observed change from one litter to the next is statistically significant, but rather that one pattern of change fits the observed data significantly better than another. Whether or not these changes in parental behavior translate into significant differences in pup bio-behavioral development is not studied here; however, the hypothesis that parity affects pup bio-behavioral development is testable.

Another major limitation of this study is its having been done with individuals exclusively from a single laboratory colony that had been outbred several generations prior from an ancestral population in natural conditions. The removal of the study population from natural environmental cues could result in patterns specific to laboratory conditions, just as could any laboratory study of this species. However, the amount of control on external conditions (e.g., *ad libitum* provisioning of food and water, no threat of predation or natural disaster, etc.) aids in our identification of this latent process of change in parental behavior that might otherwise be masked by confounding environmental factors. Finally, it should be noted that the age of the parents observed for this study is significantly older than what would be seen in the wild. It is unlikely that in the wild, pairs would live to produce more than 2 l or to begin parenting at such an advanced age. Nevertheless, given the longevity of voles in the laboratory, these findings are particularly useful for those who study the species in the laboratory setting.

## CONCLUSIONS

This study provides valuable information for our understanding of the process of parenting in a monogamous species. We find evidence, here, that prairie vole parenting is indeed a process of change, rather than a constant. Our findings support previous findings of sexual dimorphism in the parental behaviors of prairie voles, and our findings extend the conceptualization of sexual dimorphism to patterns of change in parental behaviors across litters. Moreover, we find evidence for a potentially important role of fathers in the maintenance of monogamous behavior in offspring. These results generate new hypotheses that can and should be tested with further research. Thus, we conclude that prairie vole parenting is a dynamic process of two individuals, each with their own potential to dramatically alter trajectories of bio-behavioral development in offspring. Understanding longitudinal and inter-individual dynamics of complex social behaviors and their development is critical for the informed investigation of both proximate and ultimate mechanisms that may subserve these very behaviors.

## Supplementary Material

1

2

## Figures and Tables

**TABLE 1 |:** Descriptive statistics for pup-directed behaviors, number of litters observed, and number of litters left unobserved (of the 141 total observed pairs) across litters and by litter.

Variable	Min	Mean	Max	SEM	^N^obs	^N^missing
Maternal PDB	250.0	1055.0	1522.0	14.5	303	261
Litter 1	385.0	1100.7	1489.0	21.0	122	19
Litter 2	346.2	1066.0	1508.0	24.0	95	46
Litter 3	278.3	1032.7	1380.0	32.7	53	88
Litter 4	250.0	1051.0	1522.0	53.1	33	108
Paternal PDB	0.0	628.5	1366.0	17.7	303	261
Litter 1	0.0	622.3	1218.0	26.9	122	19
Litter 2	19.0	594.1	1230.0	31.7	95	46
Litter 3	11.0	641.1	1366.0	43.8	53	88
Litter 4	15.0	730.0	1286.0	52.5	33	108

**TABLE 2 |:** Correlation coefficients for measures of pup-directed behaviors across litters (1–4) by individual (Female, X; Male, Y).

	X1	X2	X3	X4	Y1	Y2	Y3	Y4
X1	1.00							
X2	0.391	1.00						
X3	0.312	0.361	1.00					
X4	0.235	−0.033	0.257	1.00				
Y1	−0.346	0.063	0.005	−0.116	1.00			
Y2	0.028	−0.318	0.112	−0.422	0.314	1.00		
Y3	−0.059	0.081	−0.251	−0.056	0.448	0.237	1.00	
Y4	−0.440	0.014	−0.126	−0.235	0.630	0.457	0.506	1.00

**TABLE 3 |:** Indices of model fit.

Group	Model	*X*^2^	DF	P	CFI	TLI	RMSEA	AIC_*i*_	Δ_*i*_(AIC)	w_*i*_(AIC)
Mothers	No Growth	18.521	11	0.070	0.687	0.829	0.070	1400.279	3.23	0.155
	**Linear**	**9.929**	**8**	**0.318**	**0.946**	**0.960**	**0.034**	**1397.049**	**0**	**0.778**
	Non-linear	10.194	6	0.117	0.825	0.825	0.070	1401.951	4.90	0.067
Fathers	No Growth	17.246	11	0.101	0.766	0.872	0.063	1524.841	4.68	0.088
	Linear	15.021	8	0.059	0.737	0.803	0.079	4319.349	2799.20	0
	Non-linear	6.537	6	0.366	0.980	0.980	0.025	4314.865	2794.71	0
	**CNL**	**6.565**	**8**	**0.584**	**1.000**	**1.040**	**0.000**	**1520.160**	**0**	**0.912**
**Bivariate**	**Linear/CNL**	**30.616**	**28**	**0.165**	**0.935**	**0.924**	**0.044**	**2880.914**	**–**	**–**

Rows in bold indicate the model with best comparative fit (as determined through comparative fit indices) for each observed group [Mothers, Fathers, or the Dyad (Bivariate)]. Models include patterns of no growth, linear growth, non-linear growth, corrected non-linear (CNL) growth and/or a Linear/CNL Mix. Analyses of Cluster 2 are not given; for the mother, father, and dyad, the addition of covariates of litter size and parental age yielded models with exceptionally poor fit/non-converging models. In order from left to right, model fit indices presented are chi-square statistic (X^2^ ), degrees of freedom (DF), p-value (P), Comparative Fit Index (CFI), Tucker-Lewis Index (TLI), Root Mean Square Error of Approximation (RMSEA), Akaike’s Information Criterion (AIC_i_), Δ_I_(AIC), and Akaike Weights [w_i_(AIC)].
