# Supplementary material for: Longitudinal Trajectories and Inter-parental Dynamics of Prairie Vole Biparental Care
Source: Front Ecol Evol. Author manuscript; Available in PMC 2019 Aug 8. (PMC6687084; doi:10.3389/fevo.2018.00073)
Supplement: 1 [file NIHMS1000356-supplement-1.DOCX]

Supplementary Material

Longitudinal Trajectories and Inter-Parental Dynamics of Prairie Vole Biparental Care

Forrest D. Rogers^1*^, Mijke Rhemtulla^1^, Emilio Ferrer^1^, Karen L. Bales^1,2^

^1^Department of Psychology, University of California, Davis, United States

^2^California National Primate Research Center, University of California, Davis, United States

*** Correspondence:** Forrest D. Rogers: [fdrogers@ucdavis.edu](mailto:fdrogers@ucdavis.edu)

**Supplementary Table 1.** Mean number of seconds per twenty-minute (1200-second) trial spent in specific behaviors while in the home nest according to litter, including time spent in the nest, nursing, huddling (i.e. arch-back posture over pups), non-huddling contact (e.g. side-by-side contact with pups, laying on top of pups), retrieval and removal, licking and sniffing.

| Parent | Litter | In Nest | Nurse | Huddle | Non-Huddling Contact | Retrieval / Removal | Lick/Sniff |
| --- | --- | --- | --- | --- | --- | --- | --- |
| Mother | 1 | 944.4 | 954.3 | 265.3 | 5.1 | 13.1 | 120.9 |
|  | 2 | 926.3 | 952.7 | 242.3 | 5.6 | 9.1 | 98.9 |
|  | 3 | 889.7 | 899.0 | 173.9 | 7.6 | 10.7 | 94.0 |
|  | 4 | 828.5 | 866.4 | 192.8 | 4.3 | 15.4 | 120.9 |
| Father | 1 | 674.7 | -- | 101.2 | 466.4 | 3.8 | 50.8 |
|  | 2 | 636.7 | -- | 128.2 | 397.1 | 2.9 | 41.3 |
|  | 3 | 679.7 | -- | 107.7 | 485.6 | 2.3 | 39.8 |
|  | 4 | 703.5 | -- | 151.4 | 477.4 | 3.5 | 61.3 |
