# Supplementary material for: Longitudinal Trajectories and Inter-parental Dynamics of Prairie Vole Biparental Care
Source: Front Ecol Evol. Author manuscript; Available in PMC 2019 Aug 8. (PMC6687084; doi:10.3389/fevo.2018.00073)
Supplement: 2 [file NIHMS1000356-supplement-2.docx]

Supplementary Material

Longitudinal Trajectories and Inter-Parental Dynamics of Prairie Vole Biparental Care

Forrest D. Rogers^1*^, Mijke Rhemtulla^1^, Emilio Ferrer^1^, Karen L. Bales^1,2^

^1^Department of Psychology, University of California, Davis, United States

^2^California National Primate Research Center, University of California, Davis, United States

*** Correspondence:** Forrest D. Rogers: [fdrogers@ucdavis.edu](mailto:fdrogers@ucdavis.edu)

**Supplementary Figures**

The supplementary figures illustrate trajectories of individual behaviors in mothers and fathers across the four measured litters.


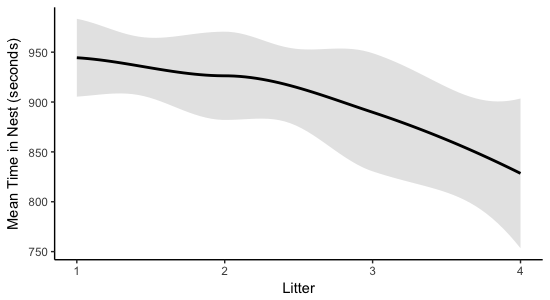


**Supplementary Figure 1.** Mean maternal time (seconds) in nest, according to litter, per 1200 second observation; mean trajectory given as thick, black line, and standard error given as gray shading.


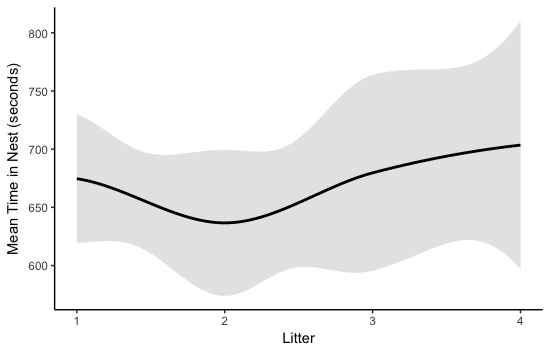


**Supplementary Figure 2.** Mean paternal time (seconds) in nest, according to litter, per 1200 second observation; mean trajectory given as thick, black line, and standard error given as gray shading.


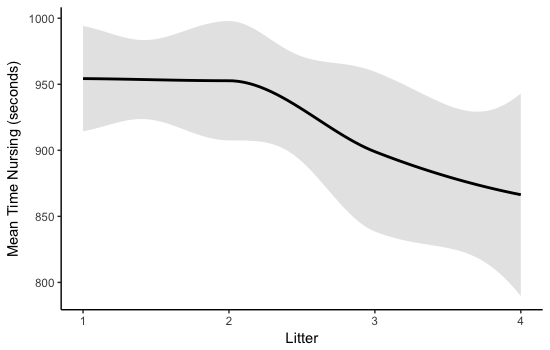


**Supplementary Figure 3.** Mean maternal time (seconds) nursing, according to litter, per 1200 second observation; mean trajectory given as thick, black line, and standard error given as gray shading.

**
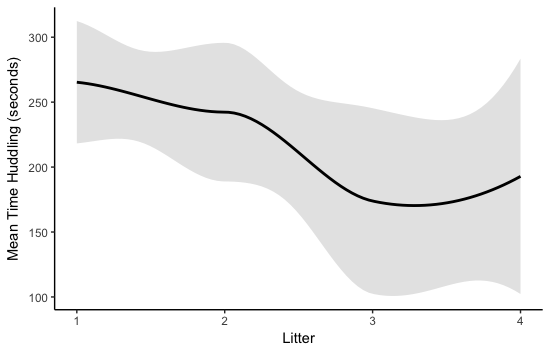
**

**Supplementary Figure 4.** Mean maternal time (seconds) huddling (i.e. arch-back nursing), according to litter, per 1200 second observation; mean trajectory given as thick, black line, and standard error given as gray shading.

**
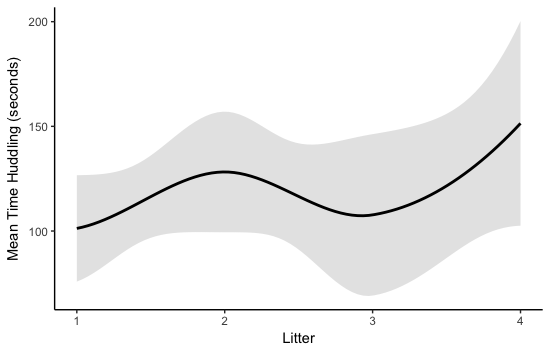
**

**Supplementary Figure 5.** Mean paternal time (seconds) huddling (i.e. arch-back nursing), according to litter, per 1200 second observation; mean trajectory given as thick, black line, and standard error given as gray shading.

**
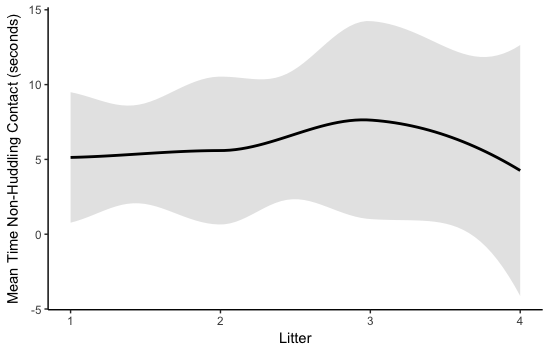
**

**Supplementary Figure 6.** Mean maternal time (seconds) in non-huddling contact, according to litter, per 1200 second observation; mean trajectory given as thick, black line, and standard error given as gray shading.

**
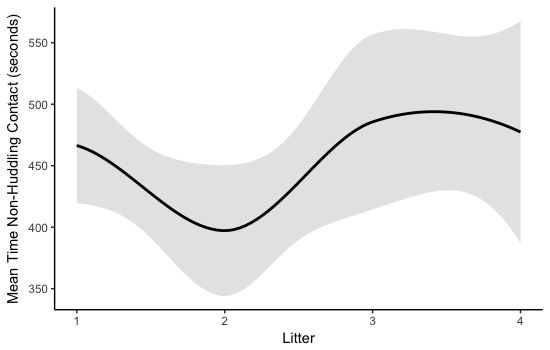
**

**Supplementary Figure 7.** Mean paternal time (seconds) in non-huddling contact according to litter, per 1200 second observation; mean trajectory given as thick, black line, and standard error given as gray shading.


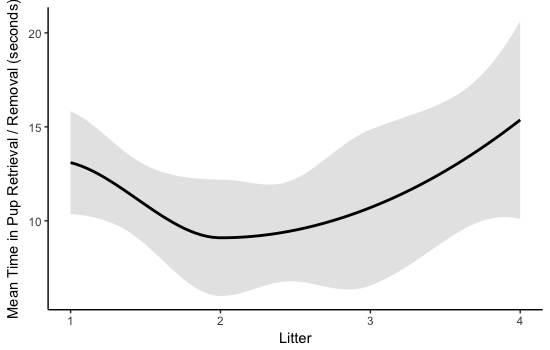


**Supplementary Figure 8.** Mean maternal time (seconds) in retrieval / removal according to litter, per 1200 second observation; mean trajectory given as thick, black line, and standard error given as gray shading.

**
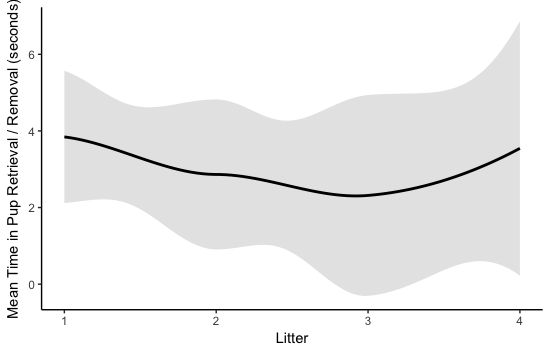
**

**Supplementary Figure 9.** Mean paternal time (seconds) in retrieval / removal according to litter, per 1200 second observation; mean trajectory given as thick, black line, and standard error given as gray shading.


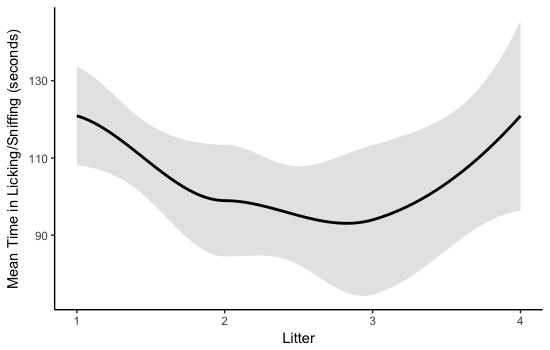


**Supplementary Figure 10.** Mean maternal time (seconds) in licking/sniffing according to litter, per 1200 second observation; mean trajectory given as thick, black line, and standard error given as gray shading.

**
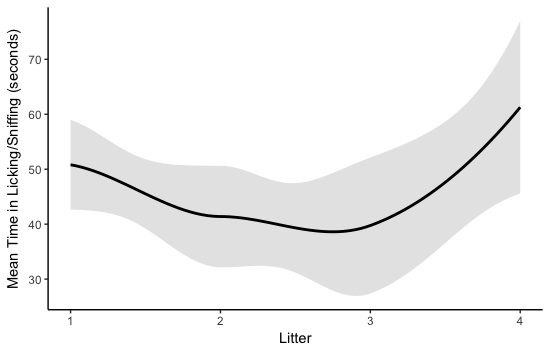
**

**Supplementary Figure 11.** Mean paternal time (seconds) in licking/sniffing according to litter, per 1200 second observation; mean trajectory given as thick, black line, and standard error given as gray shading.
